# Out-of-pocket expenditure and its correlates for institutional deliveries in private and public healthcare sectors in India: findings from NFHS 5

**DOI:** 10.1186/s12889-023-16352-w

**Published:** 2023-08-02

**Authors:** Sayantani Manna, Damini Singh, Shishirendu Ghosal, Tanveer Rehman, Srikanta Kanungo, Sanghamitra Pati

**Affiliations:** Division of Public Health, ICMR-Regional Medical Research Centre, Bhubaneswar-23, Odisha India

**Keywords:** Out-of-pocket expenditure, NFHS-5, India, Public and Private, Institutional delivery

## Abstract

**Background:**

Increased coverage for institutional delivery (ID) is one of the essential factors for improved maternal and child health (MCH). Though, ID increased over time, out-of-pocket expenditure (OOPE) for the care-seeking families had been found to be growing, parallelly. Hence, we estimated OOPE in public and private health centres for ID, along with their sources and attributing factors and compared state and union territory-wise, so that financial risk protection can be improved for MCH related services.

**Methods:**

We used women’s data from the National Family Health Survey, 2019–2021 (NFHS-5). Reproductive aged women (15–49 years) delivering one live child in last 5 years (*n* = 145,386) in any public or private institutions, were included. Descriptive statistics were presented as frequency and proportions. OOPE, was summarized as median and interquartile range (IQR). To estimate the extent for each covariate’s effect, linear regression model was conducted.

**Results:**

Overall median OOPE for ID was Rs. 4066 (median OOPE: private hospitals: Rs.25600, public hospitals: Rs.2067). Health insurance was not sufficient to slash OOPE down at private facilities. Factors associated significantly to high OOPE were mothers’ education, elderly pregnancy, complicated delivery, birth order of the latest child etc.

**Conclusion:**

A standard norm for ID should be implemented as a component of overseeing and controlling inequality. Aiding the needy is probably just one side of the solution, while the focus is required to be shifted towards reducing disparity among the health facilities, so that the beneficiaries do not need to spend on essential services or during emergencies.

**Supplementary Information:**

The online version contains supplementary material available at 10.1186/s12889-023-16352-w.

## Introduction

Since the millennium declaration, mother & child healthcare has garnered much consideration for public health policies and services among developing countries [1]. Several South-East Asian countries, including India, have significantly decreased maternal and infant mortality during the past decades [2, 3]. The improvement in maternal and child health (MCH) services were accelerated with the introduction of the National Rural Health Mission (NRHM), later modified to National Health Mission (NHM) in 2005 [4]. Cash assistance with delivery and post-delivery care for the promotion of institutional delivery (ID) was initiated in the same year (Janani Suraksha Yojana) [5]. Finally, the appointment of Accredited Social Health Activists (ASHA) to register eligible couples, pregnancies, mandatory antenatal visits of pregnant mothers followed by transportation arrangement eventually led to a gradual increase in IDs over time [6, 7].

Though ID-related services are free in public health centres, the mother’s family must pay for delivery-related services in private facilities [1]. Bills for medicines, bed charges, user fees etc., which are difficult to pay for the socially backward or financially marginal classes, force them to opt for an uncomfortable home delivery or spend a lion’s share of their income at the hospital [8]. In addition, lack of awareness to the advantages of ID, extreme geographical location, and differing cultural and social authority impact on out-of-pocket expenditure (OOPE) of the families [9, 10]. To alleviate such burdens, Janani Shishu Suraksha Karyakaram (JSSK) was launched in 2011 to provide free and cashless services to pregnant women, including transportation, diet, drugs, consumables, investigations, and cost for caesarean section and sick newborn care (up to 30 days after birth) in public health facilities [11].

Despite all these schemes and efforts, OOPE in India is one of the highest in the world [12, 13]. An earlier article based on the previous National Family Health Survey [NFHS-4, 2015–16] estimated OOPE for ID to be Rs. 5985 ($93.3) [14]. Another recent study explained how the average OOPE had mounted more than 50% over the years among most Indian states [15]. The National Health Accounts, 2018 reported that, during the financial year 2015–16, only one-third of the Total Health Expenditure (THE) was covered by public expenditure [30.6%, which was 1.18% of the Gross Domestic Products (GDP) (Rs. 1261 per capita)] and private health insurances (4.1% of THE). As a result, the citizen bears burnt of the remaining 60.6% of the health expenditure [2.3% of GDP, Rs. 2494 per capita] on their own [16]. However, purchase ability has increased for many; parallelly the choice of institute has also shifted towards private facilities, in search of availability of physician and quality care. Despite implementing several central and state policies and schemes, families still had to spend from their savings, borrow or sell assets to meet institutional expenditures. Furthermore, we have limited information on the fresh trend and interstate variation of OOPE. So, among women undergoing IDs in India, we intended to estimate the OOPE in public and private health centres, determine the associated factors influencing OOPE and compare among the states using data from the most recent and fifth National Family Health Survey (NFHS-5) 2019–2021. Additionally, we aimed to evaluate which source was more common, from where these out-of-pocket expenses were met, as a secondary objective.

## Methods

### Overview of the dataset

This study is based on the fifth round of NFHS, conducted during 2019–2021 among all the states and union territories (UTs) of India. Through this survey, information on various aspects on maternal and child health, such as fertility, infant and child mortality, the practice of family planning, maternal and child health, reproductive health, nutrition, anaemia, utilization and quality of health and family planning services etc. are collected. A holistic method of this survey [17] including selecting households and data collection procedures, has been meticulously described and published elsewhere [18].

### Sample size

**Fig. 1:**
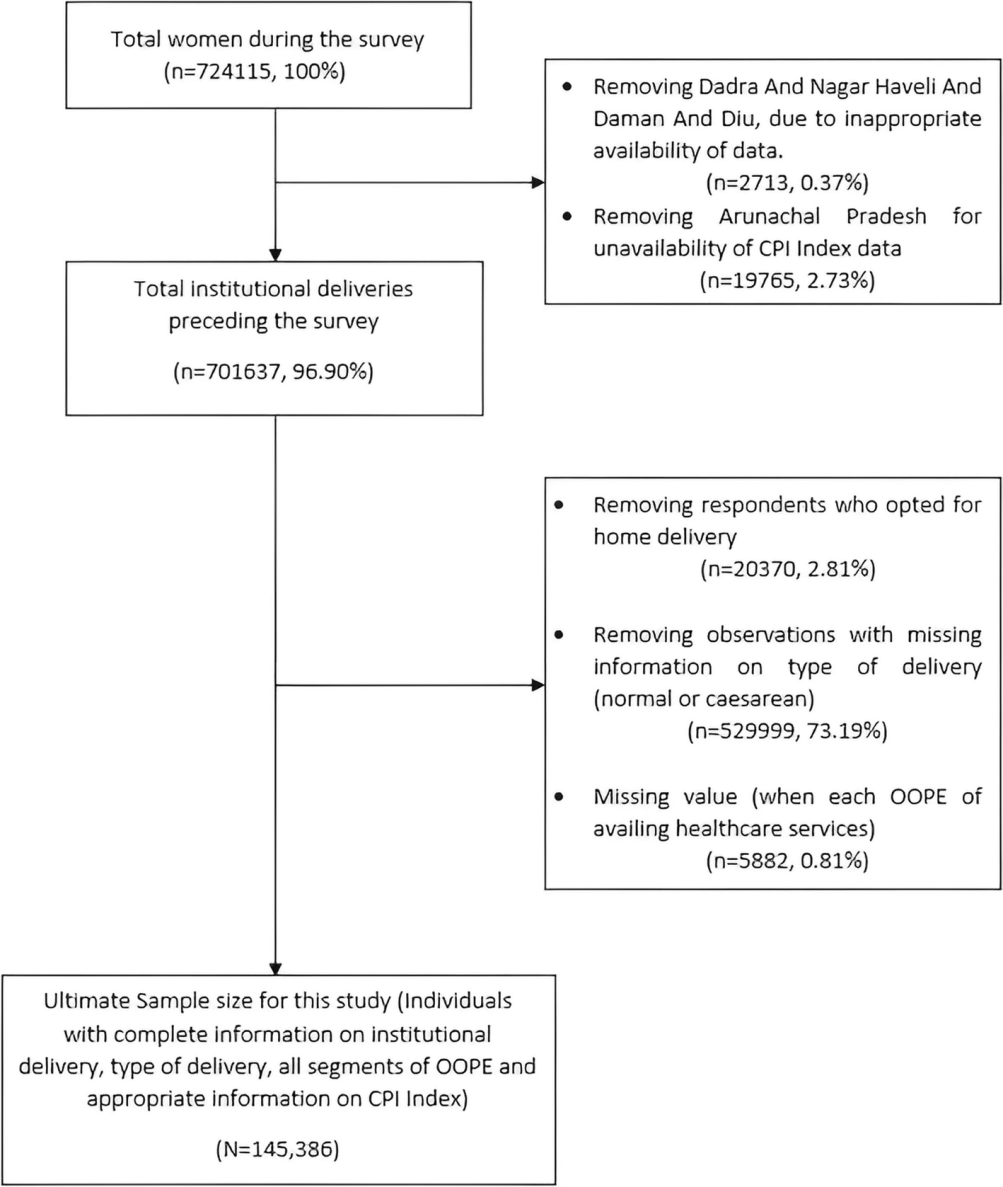
Flow diagram of sample selection from the Women’s Questionnaire of the NFHS-5

We utilized only the individual dataset, that includes information of 724,115 women of reproductive age (aged 15 to 49 years). Women experiencing motherhood during last five years from the date of interview and her delivery took place in any public or private institution, were included in this study. Among them, only the most recent delivery was considered for OOPE calculation. Women experiencing stillbirth, abortion, followed by non-IDs were excluded from the analysis. Additionally, data from one UT (Dadra and Nagar Haveli and Daman and Diu) and Arunachal Pradesh were excluded, as the data available from these areas were either unavailable or available in an unfavourable manner. Observations with missing or irrelevant data related to OOPE from any of relevant variables were left out from this analysis (Fig. 1). Ultimately, 145,386 mothers, suitable for this analysis, were included in the analysis.

### Independent variables

The mothers’ individual characteristics were explained through their age which was further categorised into four groups viz-a-viz “15 to 20 years”, “21 to 25 years”, “26 to 30 years” and greater than 30 years; place of residence; level of education attainment [no formal education, up to primary (1–5 years of schooling), junior high (6–8 years of schooling), secondary (9–10 years of schooling), higher secondary (11–12 years of schooling) and above higher secondary (> 12 years of schooling)]; ethnicity; employment status (currently employed or unemployed); living with a partner or not; asset index; and if they were covered with any health insurance (present or absent). We also divided the states and UTs into six geographical regions as east, west, north, south, central and north-east.

As supply-side factors number of ante-natal visits (< 4 visits, 4–8 visits and > 8 visits: based on old and new WHO guideline for minimum number of ante-natal visits) and type of healthcare personnel (“physicians”, “other healthcare workers” who were not doctors and “trained dai or others”) who conducted the prenatal checkups were considered as covariates of OOPE.

A few more conditions such as adolescent pregnancy (when mother’s age at the most recent birth was less than 18 years) and elderly pregnancy (mother’s age > 35 years during last live birth); complicated delivery (presence of any of the conditions during last delivery: breech presentation, prolonged labour, excessive vaginal bleeding and convulsions) and birth order of the last live birth (primigravida or not) was also considered as associated risk factors for increased OOPE.

The type institutes, where the deliveries took place, were classified into two distinct groups, public and private health facilities those included 'NGOs or Trust hospitals/clinics’. A composite variable was created combining type of institution type (public and private) and type of delivery method (caesarean and non-caesarean delivery) that represented the proportion of preference for type of delivery method and health facilities.

### Outcome variable

Outcome variable for this study was total OOPE, which was an aggregated resultant of expenditure from five distinguished variables, which are “transportation”, “hospital stay”, “laboratory tests”, “medicines” and “money paid for any other services”. Some of the individuals’ responses were recorded for each of those segments separately, while those who could not recall expenditure for each of the domains, one aggregated OOPE was recorded. Before aggregating these six variables recording OOPE, only the central 99 percentile was considered as valid, the top and lowest 0.5 percentile was considered as outliers. By adding them together one variable containing total crude OOPE was estimated.

These expenses took place at different time frames, so we converted all the expenses and made them equivalent to the financial year of 2020–2021 (April, 2020 to March, 2021). For this, first we identified state and UT wise Consumer Price Index (CPI) deflator from the financial year of 2014–15 to 2020–21 [19], as the included first observation delivered her last child to the July, 2014 and the final observation delivered during April of 2021. The total crude OOPE was then multiplied with the CPI deflator to estimate the adjusted OOPE for each individual.

### Statistical analysis

STATA 16 (Stata Corp, College Station, Texas, USA) and Excel, 2019 (Microsoft Corporation, Washington, USA) was used for data cleaning, management and statistical analysis. QGIS (v3.28) was used to prepare the graphs. Descriptive statistics of the study participants was presented as frequency with proportion. Being a continuous variable with non-normal distribution (tested through Shapiro–Wilk test), OOPE was summarized as median, followed by inter-quartile range (IQR). Before going into the multivariable linear regression model, we conducted univariable linear regression models for each possible and available covariates included for this study. Variables like health insurance, that was found insignificant from univariable regression (*p*-value: < 0.05), also causing negligible effect in multivariable model (r^2^ change < 0.01); and the variable containing information on occupation of the mothers, with greater proportion with missing values (84.8% missing value), those hamper robustness of a regression model were excluded from the final adjusted linear regression model. The NFHS sampling weights were used through the “*svyset*” command, to justify the differential probabilities of participant selection. Additionally, median OOPE irrespective of institution type (public and private institutions) and median OOPE segregated by type of institution availed were further divided into five quintiles (very high, high, medium, low and very low) to compare the magnitude of OOPE among the 34 states and UTs included in this study.

## Results

Mean age of the participants was found to be 27.3 (± 5.0) years. Among them, the majority belonged to 26–30 years age-group (36.3%), rural setup (77.1%) and unemployed (75.9%) at the time of interview (Table 1). Among them, 17.7% never received any formal education and majority were not covered under any health insurance (72.0%). A significant section of our participants reported of receiving less number of ante-natal checkups (ANC) than the standard of at least four ANC visits. Giving birth to a child before the age of 18 years was reported by 2.6% of the mother, while elderly pregnancy was evident among 3.6% of the cases. Presence of any of the signs of complicated delivery was reported by 57.6% of the mothers; prolonged labour (41.9%) and excessive vaginal bleeding (34.6%) was frequent among them.

**Table 1:** Description of the study samples (*n* = 145,386)

**Socio-demographic covariates**		**Frequency (n, %)**
**Age group**	15-20 years	9278, 6.4
	21-25 years	50308, 34.6
	26-30 years	52834, 36.3
	=31 years	32966, 22.5
**Residence**	Urban	33235, 22.8
	Rural	112151, 77.2
**Educational attainment**	No formal education	25706, 17.7
	Completed primary education Junior High	16492, 11.3 25595, 17.6
	Completed secondary education	31371, 21.6
	Higher Secondary Above Higher Secondary	21776, 14.9 24446, 16.8
**Caste**	Scheduled Caste	30055, 21.87
	Scheduled Tribe	22532, 16.4
	Other Backward Class	59049, 42.9
	None of them	25768, 18.7
**Employment**	Currently unemployed	16773, 75.8
	Currently employed	5332, 24.1
**Life Partner**	Lives without partner	2073, 1.43
	Lives with partner	143313, 98.6
**Wealth Index**	Poorest quintile	31434, 21.6
	Poorer quintile	32437, 22.3
	Middle quintile	29656, 20.4
	Richer quintile	27756, 19.1
	Richest quintile	24103, 16.6
**Region**	North	28943, 20.9
	Central	35058, 25.4
	East	25104, 18.2
	North-east	14862, 12.8
	West	13601, 9.86
	South	20386, 14.7
**Health Insurance Coverage**	Absent	104731, 72.0
	Present	40655, 27.9
**Place Of Deliveries**	Private-caesarean	19123, 13.1
	Private-normal delivery	19429, 13.4
	Public-caesarean	16608, 11.4
	Public-normal delivery	90226, 62.1
**Supply-side covariates**		**Frequency (n, %)**
**Prenatal visits**	Physicians	92495, 66.4
	Other healthcare workers, except doctors	46410, 33.4
	Trained *dai* or family members	241, 0.17
**Number of ANC visits**	<4 visits	48313, 35.1
	4-8 visits	68492, 49.8
	>8 visits	20847, 15.1
**Adolescent Pregnancy **	Yes	3791, 2.6
	No	141595, 97.4
**Elderly Pregnancy**	Yes	5230, 3.6
	No	140156, 96.4
**Pregnancy Complication**	Yes	82754, 57.6
	No	61002, 42.4
**Birth Order Number**	primigravida	52161, 35.9
	>1 baby	93225, 64.1

Overall median OOPE for ID was found to be Rs.4066 (IQR: Rs.1051–6017). Median OOPE from private health facilities [Rs.25600 (Rs.12705–46000)] was nearly 12.4 times higher than public institutions (Table 2). Overall median OOPE for the mothers from the lowest asset quintile was Rs.2200 (IQR: Rs.631–5636), which was Rs. 1771 (IQR: Rs.508–4194)] at the public institutions, while in private institutions, median individual expenditure for the same economic group was Rs.18926 (8639–34,100). Median OOPE at private health facilities was higher for those with health insurance [median OOPE: Rs. 26,477 (IQR: Rs.12868–47,194)], than those mothers who were not covered under any health insurance [median OOPE: Rs.25434 (IQR: Rs.12682–45,655)]; though this scenario was just the opposite in public facilities [median OOPE: Rs.1938 for those mothers covered under health insurance vs Rs.2067 for those not covered with health insurance]. Requirement or opting for caesarean section drastically increased the median OOPE in both private and public institutions. Mothers receiving the optimum number of ANC visits (4 to 8 ANC visits) had paid least at the public health facilities median OOPE: Rs.1860 (IQR: Rs.365–4932)], than the other two groups. In case of elderly pregnancy, the median OOPE was found to be on the higher note than adolescent pregnancy in both public and private institutions. Complicated delivery and primigravida were two other conditions where median OOPE were estimated to be on the higher side, irrespective of institution type.

**Table 2:** Median out-of-pocket expenditure from institutional deliveries due to various covariates

**Covariates**	**Private Institution (Median, IQR)**	**Public Institution (Median, IQR)**	**Overall (Median, IQR)**
**Overall OOPE incurred due to institutional delivery care services**	25600 (12705, 46000)	2067 (503, 5082)	4066 (1051, 6017)
**Age group (*n*=145,386)**			
15-20 years	25339 (1735, 42792)	2067 (476, 5167)	3215 (800, 11481)
21-25 years	24659 (12197, 44286)	2095 (525, 5087)	3686 (1009, 13592)
26-30 years	25833 (12917, 45926)	2021 (477,5027)	4189 (101, 17092)
≥ = 31 years	27026 (13175, 47898)	2019 (489, 5167)	5027 (1163, 21114)
**Residence (*n*=145,386)**			
Urban	28417 (14249, 49344)	2067 (354, 5636)	7294 (1409, 27026)
Rural	23914 (11689, 43400)	2039 (525, 4928)	3358 (930, 11941)
**Educational attainment (*n*=145,386)**			
No formal education	17222 (8267, 32669)	1722 (489, 4079)	2340 (702, 6678)
Primary education	19554 (9864, 37620)	1754 (433, 4357)	2514 (689, 7863)
Junior High	21257 (10508, 38706)	1832 (354, 4709)	2906 (709, 10146)
Secondary education	25306 (13053, 44563)	2377 (564, 5918)	4493 (1148, 15738)
Higher Secondary	26271 (13195, 45366)	2310 (517,5741)	5564 (1292, 21017)
Above Higher Secondary	31689 (16377, 52462)	2531 (664, 6305)	13778 (2541, 37526)
**Social classes (*n*=137,404)**			
Scheduled Tribe	22963 (11139, 41877)	1973 (517, 4822)	3049 (838, 10521)
Scheduled Caste	21227 (11022, 41492)	1409 (9, 3945)	2022 (302, 7045)
Other Backward Class	25779 (12682, 46436)	2117 (574, 5127)	4509 (1170, 17895)
None of the casts	28182 (14183, 49763)	2259 (486, 5882)	7186 (1468, 26137)
**Employment (*n*=22,105)**			
Currently unemployed	25833 (13036, 46057)	2067 (525, 5167)	4200 (1063, 16346)
Currently employed	24862 (12917, 44751)	2037 (508, 4960)	3351 (816, 12917)
**Life Partner (*n*=145,386)**			
Lives without partner	25833 (11766, 45655)	1943 (539, 5221)	3400 (845, 13053)
Lives with partner	25595 (12705, 46011)	2067 (503, 5082)	4079 (1051, 16064)
**Wealth Index (*n*=145,386)**			
Poorest quintile	18926 (8639, 34100)	1771 (508, 4194)	2200 (631, 5636)
Poorer quintile	20328 (9789, 37805)	2067 (531, 5027)	2870 (816, 8857)
Middle quintile	23795 (11481, 42341)	2214 (544, 5511)	3974 (1051, 13668)
Richer quintile	26231 (13373, 46970)	2255 (489, 5787)	6302 (1409, 2284)
Richest quintile	30300 (15500, 51667)	2114 (211, 5778)	14249 (2340, 37200)
**Region (*n*=137,954)**			
North	24542 (12449, 43772)	1632 (0, 4593)	3153 (539, 13286)
Central	21017 (9859, 41036)	1292 (203, 3358)	2312 (525, 9789)
East	25436 (12718, 42160)	2480 (838, 5314)	3577 (1240, 11341)
North-east	33762 (14395, 56082)	5044 (2207, 10145)	5800 (2492, 13386)
West	23673 (12400, 45091)	1409 (113, 4537)	7186 (1127, 23560)
South	31709 (17149, 51814)	3179 (1127, 7074)	8267 (2175, 28654)
**Health Insurance Coverage (*n*=145,386)**			
Absent	25434 (12682, 45654)	2067 (517, 5073)	4165 (1063, 16316)
Present	26477 (12868, 47194)	1938 (408, 5167)	3720 (912, 15018)
**Type Of Delivery Method Opted (*n*=** **145,386)**			
Normal delivery	17169 (9435, 29884)	1786 (413, 4326)	2696 (721, 8455)
Caesarean delivery	37805 (21510, 58649)	4429 (1196, 10941)	20132 (4559, 44433)
**Supply-side covariates**			
** Prenatal visits (*n*=139,146)**			
Physicians	27280 (13761, 47814)	2312 (571, 5711)	5439 (1305, 21000)
Other healthcare workers, except doctors	21136 (10333, 39455)	1632 (326, 4043)	2431 (631, 8267)
Trained dai or family members	25686 (16056, 44286)	2102 (636, 6434)	5073 (1148, 15933)
**Number of ANC visits (*n*=137,652)**			
<4 visits	20925 (10266, 40656)	2033 (525, 4895)	3363 (946, 11446)
4-8 visits	26231 (13354, 46293)	1860 (365, 4932)	4043 (943, 16898)
>8 visits	32148 (17050, 51946)	2543 (705, 6327)	7209 (1691, 28464)
**Adolescent Pregnancy (*n*=145,386)**			
Yes	24313 (12664, 42034)	1908 (425, 5054)	3024 (756, 11071)
No	25655 (12705, 46095)	2067 (508, 5082)	4102 (1051, 16262)
**Elderly Pregnancy (*n*=145,386)**			
Yes	28546 (12283, 51667)	1837 (489, 4627)	3993 (973, 19000)
No	25529 (12705, 45738)	2067 (505, 5087)	4066 (1051, 15921)
**Pregnancy Complication (*n*=143,756)**			
Yes	26009 (12964, 46500)	2175 (544, 5391)	4213 (1113, 16174)
No	24800 (12042, 45091)	1832 (407, 4593)	3701 (925, 15237)
**Birth Order Number (*n*=145,386)**			
Primigravida	28909 (14588, 50579)	2303 (508, 5849)	5623 (1257, 22682)
>1 baby	23439 (11573, 42402)	1908 (503, 4769)	3444 (925, 12718)

The strength of association for each covariate was estimated separately through the univariable models (Supplementary Table S1). The multivariable model showed, burden of OOPE substantially impact on mothers living in rural setup (ß-coefficient: 372.2, 95% CI: -79.8 to 824.2), higher education (ß-coefficient for above higher secondary education: 1972.8, 95% CI: 1343.9 to 2601.7), not belonging to any of the designated ethnic group (ß-coefficient: 1641.1, 95% CI: 1030.7 to 2251.4), belonging to the highest wealth quintile (ß-coefficient: 2118.0, 95% CI: 1452.3 to 2783.7) and living in the region of North-East (ß-coefficient: 5314.6, 95% CI: 4469.6 to 6159.5) (Table 3). Though insignificantly, due to very small proportion, prenatal checkups through the trained *dai*s led to very high OOPE (ß-coefficient: 6657.0, 95% CI: -5562.2 to 18,876.3) as compared to checkups conducted by nurses, ANM or ASHA workers. Complicated delivery increased the OOPE up by Rs.823.9 (95% CI: 482.3 to 1165.6) than uncomplicated deliveries. Similar finding was evident in case of primigravida, where the OOPE was higher by Rs.1316.5 (95% CI: 912.5 to 1720.5) than order of delivery is second or more. Compared to the non-caesarean deliveries at public institutions, likelihood for higher OOPE for private-caesarean was highest (ß-coefficient: 39,659.6, 95% CI: 38,835.4 to 40,483.8), followed by private non-caesarean (ß-coefficient: 18,224.0, 95% CI: 17,712.6 to 18,735.3) and caesarean deliveries at public institutions (ß-coefficient: 4208.0, 95% CI: 3828.1 to 4587.9).

**Table 3:** Association of out-of-pocket expenditure from institutional deliveries with various socio-demographic and supply-side covariates along with risk factors of complicated delivery

Characteristics	ß-coefficient (95% CI)
**Residence**	
Urban	Reference
Rural	372.20 (79.82, 824.24)
**Educational attainment**	
No formal education	Reference
Completed primary education	181.63 (-209.42, 572.69)
Junior High	-63.35 (-431.71, 305.01)
Completed secondary education	282.70 (-136.36, 701.76)
Higher Secondary	586.92 (64.74, 1109.10)
Above Higher Secondary	1972.76 (1343.87, 2601.67)
**Social classes**	
Scheduled Tribe	Reference
Scheduled Caste	-220.39 (-669.37, 228.59)
Other Backward Class	636.25 (147.81, 1124.68)
None of the casts	1641.05 (1030.67, 2251.43)
**Wealth Index**	
Poorest quintile	Reference
Poorer quintile	-145.01 (-457.05, 167.02)
Middle quintile	-242.86 (-650.22, 164.50)
Richer quintile	346.02 (-143.89, 835.92)
Richest quintile	2118.01 (1452.34, 2783.69)
**Region**	
North	Reference
Central	-757.78 (-1181.55, -334.01)
East	378.42 (-110.99, 867.83)
North-east	5314.55 (4469.60, 6159.50)
West	319.33 (-297.33, 935.99)
South	2402.92 (1678.24, 3127.60)
**Prenatal Visits**	
Physicians	520.17 (230.59, 809.74)
Other healthcare workers, except doctors	Reference
Trained dai or family members	6657.06 (-5562.18, 18,876.31)
**Number of ANC visits**	
< 4 visits	Reference
4–8 visits	368.70 (45.75, 691.64)
> 8 visits	494.21 (-90.02, 1078.43)
**Adolescent Pregnancy**	
Yes	-757.10 (-1555.89, 41.70)
No	Reference
**Elderly Pregnancy**	
Yes	1283.64 (210.88, 2356.40)
No	Reference
**Pregnancy Complication**	
Yes	823.94 (482.26, 1165.62)
No	Reference
**Birth Order Number**	
Primigravida	1316.50 (912.49, 1720.51)
> 1 baby	Reference
**Place Of Deliveries**	
Private-caesarean	39659.61 (38835.40, 40483.82)
Private-normal delivery	18223.95 (17712.57, 18735.33)
Public-caesarean	4207.98 (3828.11, 4587.85)
Public-normal delivery	Reference

When it came to the source of paying hospital bills, savings in bank was one of the favourable sources in most of the instances (84.8%), followed by borrowing from known ones (17.5%). A detailed description of the same with median amount of OOPE met from each souces has been presented through the Table 4a and b.

**Table 4:** Description of various sources and median amount paid from each source for institutional delivery and different delivery method

**a: Financial source to meet OOPE for the most recent delivery**			
Characteristics	n	%	95% CI of %
**OOPE met through bank savings [*n*** **= 108503; 84.83%]**			
Private-caesarean	19135	84.93	84.45–85.39
Private-normal delivery	19632	87.56	87.12–87.99
Public-caesarean	11974	84.29	83.69–84.89
Public-normal delivery	57762	84.02	83.75–84.29
**OOPE met by borrowing from friends [*n*** **= 22327; 17.46%]**			
Private-caesarean	5588	24.80	24.39–25.37
Private-normal delivery	4125	18.40	17.89–18.91
Public-caesarean	2343	16.50	15.88–17.15
Public-normal delivery	10270	14.94	14.67–15.21
**OOPE met by selling property [*n*** **= 2219; 1.74%]**			
Private-caesarean	425	1.89	1.71–2.07
Private-normal delivery	335	1.49	1.34–1.66
Public-caesarean	231	1.63	1.43–1.85
Public-normal delivery	1228	1.79	1.69–1.89
**OOPE met by selling jewellery [*n*** **= 2130; 1.67%]**			
Private-caesarean	751	3.34	3.11–3.58
Private-normal delivery	377	1.68	1.52–1.86
Public-caesarean	295	2.08	1.85–2.32
Public-normal delivery	706	1.03	00.95–1.11
**OOPE met from health insurance & any other sources [*n*** **= 4971; 3.89%]**			
Private-caesarean	1058	4.70	4.42–4.98
Private-normal delivery	639	2.85	2.64–3.10
Public-caesarean	611	4.30	3.97–4.64
Public-normal delivery	2664	3.87	3.73–4.02
**b: Median amount paid from different sources**			
Characteristics	n	Median (Rs.)	Inter-quartile range (Rs.)
**OOPE met through bank savings**			
Private-caesarean	16135	38027	22000, 58491
Private-normal delivery	16695	17714	10062, 30380
Public-caesarean	12828	5439	2175, 12,000
Public-normal delivery	63575	2447	1148, 5087
**OOPE met by borrowing from friends**			
Private-caesarean	4848	40656	24261, 61718
Private-normal delivery	4125	18123	10381, 31000
Public-caesarean	2601	8211	3974, 18,600
Public-normal delivery	11148	3619	1691, 7154
**OOPE met by selling property**			
Private-caesarean	379	41551	19419, 67865
Private-normal delivery	327	18318	8881, 31,795
Public-caesarean	317	5741	2191, 11273
Public-normal delivery	1399	2480	939, 5669
**OOPE met by selling jewellery**			
Private-caesarean	617	44396	26867, 63409
Private-normal delivery	311	27837	17022, 48279
Public-caesarean	258	10222	4608, 20667
Public-normal delivery	704	4079	1786, 8037
**OOPE met from health insurance & any other sources**			
Private-caesarean	815	38592	19688, 65351
Private-normal delivery	551	18235	8928, 33114
Public-caesarean	539	5191	1824, 13109
Public-normal delivery	2467	2296	912, 5087

**Fig. 2:**
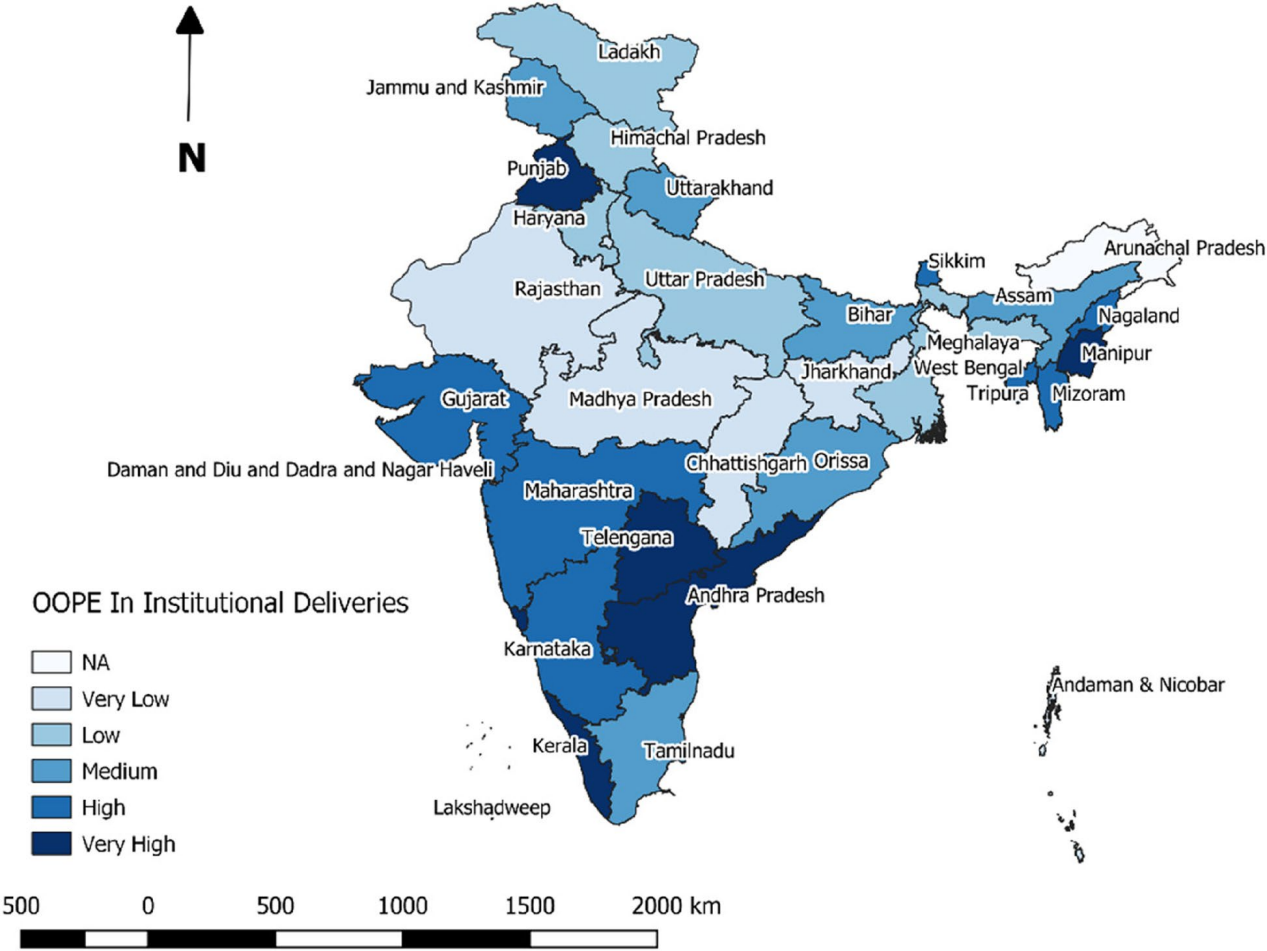
State and UT-wise distribution of median OOPE for institutional deliveries

**Fig. 3:**
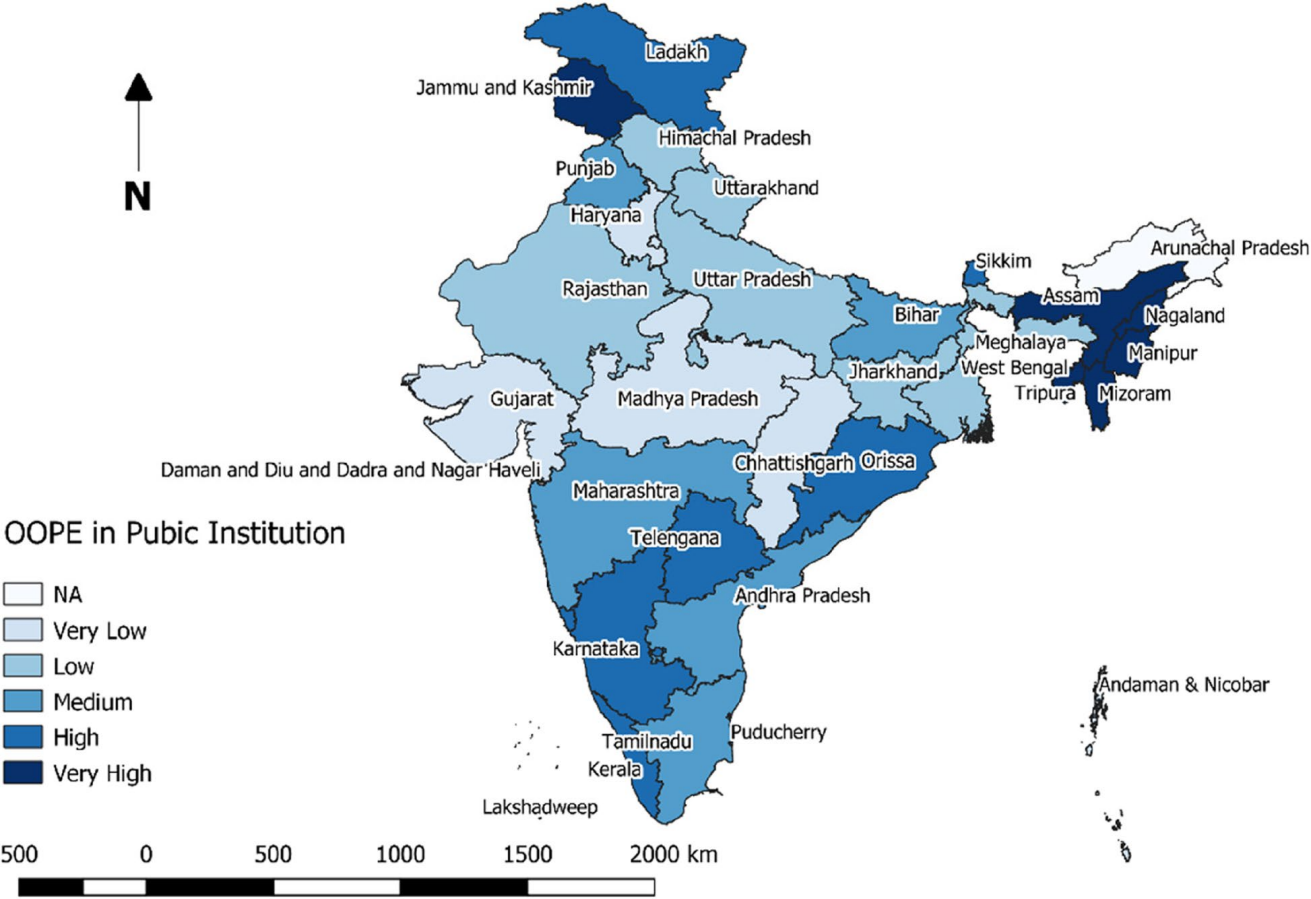
State and UT-wise distribution of median OOPE for deliveries at public institutions

**Fig. 4:**
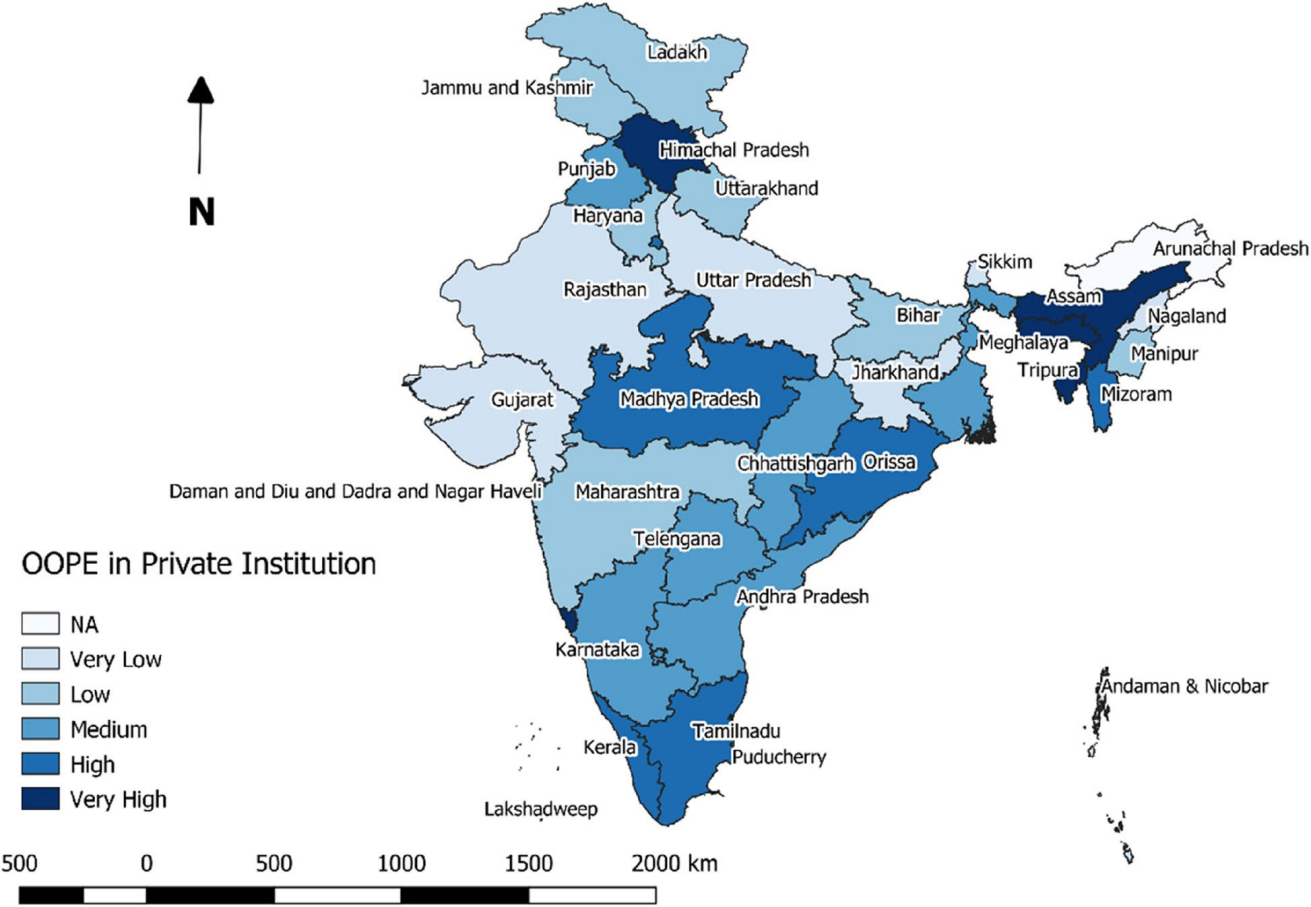
State and UT-wise distribution of median OOPE for deliveries at private institutions

Highest median OOPE for ID was reported from several southern states, such as, Kerala (Rs.20667), Telengana (Rs.11689); while minimum OOPE was reported from Madhya Pradesh (Rs.886) (Fig. 2). When separated by the type of institution availed, five from the North- Eastern states (Manipur, Tripura, Mizoram, Assam and Nagaland) topped the list in terms of OOPE for ID at public institutions (Fig. 3). Least median OOPE from private institutions was estimated from Andaman & Nicobar (Rs.9688) followed by Sikkim (Rs.10646), while highest median OOPE at private hospitals was reported from Lakshadweep (Rs.48627), another two states from Nort-East [Meghalaya (Rs.41333) and Assam (Rs.36254)] was among the top five states in terms of OOPE for ID at private hospitals (Fig. 4).

## Discussion

This article provides an insight towards the state-specific and union territory-wise OOPE for IDs from India's public and private health sectors. After adopting several initiatives in favour of reducing personal expenses, still OOPE for IDs was found to be on the high note, even at public health facilities. OOPE for IDs at private health facilities was higher than personal spending at public institutions among all socio-demographic strata. Other noteworthy findings from this study were, women with higher educational attainment had highest OOPE for both type of IDs than other level of educational attainment; more in private than public ones. Secondly, elderly pregnancy had more influence on increased OOPE than adolescent pregnancy. Also, health insurance coverage didn’t protect from excessive OOPE on delivery at private health facilities. And probably, high proportion of complicated deliveries increased the median OOPE to this high.

Cleanliness, quality and rapid services, personal care, better doctor/nurse-to-patient ratio: such conceptions drive general people towards private institutions [20]. Day-by-day, cost of availing health care is increasing [20], so is willingness and ability to purchase, which steer a lofty chunk of our population in the direction to high out-of-pocket spending at private institutions [21]. OOPE was found to be more from both rural and urban private institutions, however, the median OOPE was less at rural private health facilities; probably due to pregnant women and/or their families were unable to avail healthcare services, so opting more for public institutions. Other possible reasons could be lesser rate for the same services at rural setups, which is generally influenced by local economic flow.

The median OOPE was also found to be increasing with mothers’ educational attainment, which was highest among women with the highest level of schooling. Such a similar trend was also evident among the wealth quintiles. In Indian scenario, there is a positive correlation between education and wealth, which helps women to be in school for a longer period [22]. This provide them with information related to adolescent health and hygiene, sexual understanding, child care etc. [23], which could be a possible reason for opting private health centres for delivery, shelling out more coins from their pockets. Also, just belonging to higher socio-economic status, demands better healthcare services for which those mothers and/or their families are generally capable of paying [24].

Delayed childbearing, driven by factors like educational and career pursuits, has become more common [25]. However, the biological factors, responsible for conceiving a child, are to be acknowledged too. With increasing age, women experience age-related fertility decline and an increased risk of pregnancy complications [25]. This contributes to the greater healthcare costs associated with elderly pregnancies. Advanced maternal age necessitates additional medical interventions and monitoring during pregnancy and delivery due to the increased likelihood of age-related health issues. In case of elderly pregnancy, conditions like gestational diabetes, hypertension, placental abnormalities, and caesarean sections are more prevalent, leading to increased expenses for their treatment. Complications during delivery result in longer hospital stays, postpartum care, and follow-up visits, all contributing to higher out-of-pocket expenses. Such conditions are associated with a greater risk of obstetric and neonatal complications, requiring specialized care, emergency interventions, extended hospital stays, and additional postpartum care. Insurance coverage may not include all aspects of care, leaving individuals responsible for a larger share of expenses [26]. The extensive healthcare interventions, including advanced prenatal care, specialized tests, consultations, and closer monitoring, lead to higher healthcare costs. Specialized healthcare providers, facilities, or technologies may also be required, further increasing out-of-pocket expenses.

If we look into the disadvantages of visiting a private health facility for ID, some of the prominent elements comes out like: very few private health insurances provide coverage for ID at private health facilities [27], not all; even if they cover, few of the services like bed/cabin charge are covered. Moreover, private institutions have a tendency for choosing surgical intervention over normal delivery [28], which is chosen by a greater number of mothers due to relatively less painful and stressful delivery, especially, while giving birth to her first child[29].To overcome such issues, the NHM in India had been working for over 15 years aiming to increase service coverage, maximising equity in health sectors and health outcomes on one hand; while, exclusively aimed to minimize OOPE and catastrophic health spending specifically among the deprived, disadvantaged and most vulnerable groups on the other hand [27]. But the problem lies in higher proportion of private institutions (around 70%) in the Indian health system and many small institutions are adding up to the list in a faster rate than enrolling them under existing health insurance schemes. In this regard, suggestion would be to make it obligatory for all health facilities (both public and private) to provide adequate maternity services at no cost or at a subsidised rate, so that the root objective of the NHM, which is aimed to achieve universal health coverage [30] can be achieved. Besides that, both the rate of acceptability and range of maternal services at both private and public health facilities are to be increased so that the current gap can be minimised.

To increase the number of ID and lessen the OOPE, several schemes were launched under the stewardship of the Ministry of Health and Family Welfare, Govt. of India. The Janani Suraksha Yojana (JSY) advocated increase in institutional births and decrease in OOPE. The JSSK entitles all normal and caesarean delivery related services including drugs, consumables, laboratory tests, transportation including referral to other public facilities and blood for no cost to all pregnant women delivering in public health facilities [31, 32]. An earlier study indicated poor utilization of JSY by the most deprived and less educational attainment, that calls for special attention to the vulnerable groups [31]. Moreover, state specific ad hoc programs like Chiranjeevi Schemes in Gujarat [33] and ACCORD in Tamil Nadu [34], opened more possibilities towards bringing down OOPE. Similar initiative by the Odisha government was named “Mamata”, that also included partial wage compensation for the mothers, so that they can have adequate rest, post-delivery [34].

### Strength & limitations

We conducted this study on the fifth edition of the NFHS, which is a nationally representative study, proving not only national level insights, but regional and state-level understanding too. The scientifically calculated robust sample size adds sufficient power to generalizability of the study outcomes. As we utilised the method of cross-sectional studies, plausibility cannot be established like in longitudinal data. Recall bias and reporting errors might be associated with age, years of completed education. Besides, the OOPE mentioned by the respondents might not be as accurate as the original; as a result, the OOPE calculated might be either under- or over-estimated.

### Implementations/suggestions

Based on the findings, it can be suggested that financial incentives under central government schemes should be promoted to attract for IDs in public sectors. This will enhance to reduced OOPE as well. These financial aids are also needed to be revised regularly to keep their eminency alive. Adding ID under financial protection efforts and complementing more private institutions under the umbrella of public as well as maximum number of private insurance schemes would facilitate minimum personal expenditure. Intensive vigilance and strict action against malpractices, like diverting needy patients to private care, excessive withdrawal from insured families etc. [28]. will help the families of disregarded mothers to restrict OOPE. Furthermore, it is required to remodel the public facilities to accommodate the growing population, as well as to regulate the cost of various maternal and child health related services at private institutions, based on their location. Last but not the least, by encouraging natural birth over caesarean section could reduce the cost paid by many folds [20].

## Conclusion

Despite several initiatives by the central government and local governing bodies, OOPE for IDs were substantially elevated from both private and government aided health facilities. As an accessible, equitable, quality health service is right to every citizen, proper healthcare during birth giving is a mother’s right. Promoting institutional delivery is essential to provide that quality health facilities to all expecting mothers, but, at the same time putting her and her family into financial distress could not be a barrier to receive maternal services. It is the high time for, advisers, implementers to the policy makers, to join hand, bring transformation to the existing funding facilities in healthcare to remove the remaining inequity so that mothers and their family can receive the benefit in an unperturbed way in the coming days.

## Supplementary Information

**Additional file 1.**

## Data Availability

The dataset generated during and/or analysed during the current study is available from the Demographic and Health Surveys (DHS) repository (with proper permission), Available at: https://www.dhsprogram.com/data/dataset/India_Standard-DHS_2020.cfm?flag=0.

## Abbreviations

NFHS: National Family Health Survey
OOPE: Out-of-Pocket Expenditure
MCH: Maternal and Child Health
ID: Institutional Delivery
NHM: National Health Mission
JSY: Janani Suraksha Yojana
JSSK: Janani Sishu Suraksha Karyakaram
