# Supplementary material for: Out-of-pocket expenditure and its correlates for institutional deliveries in private and public healthcare sectors in India: findings from NFHS 5
Source: BMC Public Health. 2023 Aug 2;23:1474. doi: 10.1186/s12889-023-16352-w (PMC10398927; doi:10.1186/s12889-023-16352-w)
Supplement: Supplementary file 1 — Additional file 1. [file 12889_2023_16352_MOESM1_ESM.docx]

**Detailed method to calculate Adjusted OOPE.**

**Calculating crude OOPE**

The NFHS-5 data recorded OOPE on child birth for the second time. The data for OOPE is of comprehensive value; using the same without any change might lead to underestimation of OOPE, as every year due to inflation, the cost of availing any service increases in absolute value (here in Rupee).

In the survey schedule, participants were given two options, either they could respond the total OOPE occurred during the last birth, or they could respond for, how much was the spending on transportation, hospital stay, medicines, tests required and any other expenses related to the delivery process separately. Some responses were recorded as 99998 which was the code for “Do Not Know” and some responses were found to be missing (reported as “**.**” in the dataset). If 99998 or “**.**” was coded for all the six variables, that observation was removed. Now, each of the six variables were tested for outliers; the upper and lower 0.5^th^ percentile were converted to missing data for each variable indicating for OOPE. In the next step, all the six variables were added with the “*egen*” command to estimate the total crude OOPE, so that none of the observations left behind.

**Adjusted OOPE**

As the reported out of pocket expenditure, for the last birth, occurred at different time points, the OOPE is required to be adjusted to a constant price, that considers inflation over the time, in order to make it comparable among the states and UTs.

The Consumer Price Index (CPI) scores for every state and UT are released every financial year (April to March of consecutive year) by the Indian government since 2011 [20]. From July 2014 through April 2021 (time period of last live birth, reported by the study participants), we used the annual CPI of each state and UT and that value was used as the denominator for identifying the deflator value for that state or UT, for a particular year (formula 1).

The CPI deflator value for *i*^th^ year= CPI of 2021/CPI in *i*^th^ year ……………………….… (1)

*(i*^th^ year: such as 2014-15, 2015-16, ..., 2020-21)

The child’s month and year of birth was the indicator for choosing the CPI deflator value for that particular state/UT and that financial year. For example, we considered CPI deflator for the financial year of 2014 for the births recorded during July, 2014 to March, 2015. Similarly, births during April, 2015 to March, 2016 was adjusted with the CPI deflator calculated for the financial year of 2015. The NFHS-5 survey recorded reports on delivery till April, 2021, which belonged to the financial year of 2021. For that one month (April, 2021), the OOPE was considered as it was made available in the dataset, as the CPI deflator for that month was 1 (one) (from the formula 1).

In the next step, the unadjusted total OOPE was multiplied to the unique deflator value of the child’s birth year and the province where he/she was born. This aided to provide comparable OOPE estimates for delivery care in India.

**Notes**

1. CPI data for the state of Arunachal Pradesh was not available from the verified source. As a result, OOPE of the aforementioned state was not calculated [20].
2. For calculating State-wise Consumer Price Index (CPI) inflation in 2020-21 the average CPI Index for ten months has been taken due to unavailability of data for the months of April and May 2020 [20]. Due to this reason, RBI calculated CPI for 2020 based on the data available from June 2020 to March 2021, which was used in our analysis for the observations who delivered during the month of April, 2021.
3. CPI score for Ladakh was not available separately, so the average of all UTs was used.
4. Earlier Daman and Diu, and Dadra and Nagar Haveli were separate UTs, so separate CPI scores were provided by the RBI [20]. But on 26^th^ January 2020, these two UTs were merged to one. As a result, it became difficult to estimate the OOPE from two different CPI scores, so were removed from the analysis.

**Supplementary table S1: Univariable association of various covariates with OOPE from institutional delivery.**

| Characteristics | **β-coefficient** | 95% CI |
| --- | --- | --- |
| **Residence** | | |
| Urban | Reference | |
| Rural | -7304.67 | -7867.11, -6742.23 |
| **Education** | | |
| No formal education | Reference | |
| Completed primary education | 1252.98 | 817.43, 1688.53 |
| Junior High | 2915.82 | 2501.669, 3329.973 |
| Completed secondary education | 6231.72 | 5771.22, 6692.22 |
| Higher Secondary | 8994.90 | 8414.07, 9575.73 |
| Above Higher Secondary | 17760.03 | 17105.05, 18415.01 |
| **Caste** | | |
| Scheduled Tribe | Reference | |
| Scheduled Caste | 2408.35 | 1856.19, 2960.52 |
| Other Backward Class | 6784.69 | 6229.75, 7339.63 |
| None of the casts | 11051.23 | 10325.08, 11777.38 |
| **Life Partner** | | |
| Lives without partner | -1996.49 | -3292.24, -700.75 |
| Lives with partner | Reference | |
| **Wealth Index** | | |
| Poorest quintile | Reference | |
| Poorer quintile | 2867.57 | 2530.87, 3204.27 |
| Middle quintile | 6228.16 | 5802.19, 6654.14 |
| Richer quintile | 10808.85 | 10319.22, 11298.49 |
| Richest quintile | 18807.86 | 18116.31, 19499.41 |
| **Region** | | |
| North | Reference | |
| Central | -1922.55 | -2408.86, -1436.24 |
| East | -1245.103 | -1788.66, -701.54 |
| North-east | 673.26 | -78.75, 1425.29 |
| West | 5045.65 | 4182.31, 5908.99 |
| South | 8435.39 | 7656.52, 9214.26 |
| **Health Insurance Coverage** | | |
| Absent | 233.22 | -227.96, 694.42 |
| Present | Reference | |
| **Prenatal Visits** | | |
| Physicians | 7281.32 | 6911.19, 7651.46 |
| Other healthcare workers, except doctors | Reference | |
| Trained dai or family members | 9319.83 | -2830.97, 21470.63 |
| **Number of ANC visits** | | |
| <4 visits | Reference | |
| 4-8 visits | 3346.42 | 2955.28, 3737.57 |
| >8 visits | 8269.36 | 7618.39, 8920.33 |
| **Early Pregnancy** | | |
| Yes | -3735.35 | -4568.66, -2902.04) |
| No | Reference | |
| **Delayed Pregnancy** | | |
| Yes | 2295.27 | 1031.58, 3558.96 |
| No | Reference | |
| **Complicated delivery** | | |
| Yes | 512.41 | 101.58, 923.23 |
| No | Reference | |
| **Birth order** | | |
| Primigravida | 5252.24 | 4830.01, 5674.48 |
| >1 baby | Reference | |
| **Institution and type of delivery** | | |
| Private-caesarean | 41389.42 | 40632.87, 42145.98 |
| Private-normal delivery | 19656.77 | 19158.89, 20154.66 |
| Public-caesarean | 5724.33 | 5411.55, 6037.11 |
| Public-normal delivery | Reference | |
